# Omicron Variant of SARS-CoV-2 Virus: *In Silico* Evaluation of the Possible Impact on People Affected by Diabetes Mellitus

**DOI:** 10.3389/fendo.2022.847993

**Published:** 2022-03-07

**Authors:** Davide Bassani, Eugenio Ragazzi, Annunziata Lapolla, Giovanni Sartore, Stefano Moro

**Affiliations:** ^1^ Department of Pharmaceutical and Pharmacological Sciences (DSF), Molecular Modeling Section (MMS), University of Padova School of Medicine and Surgery, Padua, Italy; ^2^ Department of Pharmaceutical and Pharmacological Sciences (DSF), University of Padova School of Medicine and Surgery, Padua, Italy; ^3^ Department of Medicine (DIMED), University of Padova School of Medicine and Surgery, Padua, Italy

**Keywords:** COVID-19, diabetes mellitus, glycation, human ACE2 receptor, Maillard reaction, Spike protein

## Abstract

The Omicron variant of SARS-CoV-2 (Spike mutant B.1.1.529) carrying more than 30-point mutations in its structure, of which 15 are localized in the receptor-binding domain (RBD), allows to hypothesize a relevant change in interactivity with ACE2. In previous reports we hypothesized that the worse outcome of the COVID-19 disease in diabetes mellitus condition could be related to the non-enzymatic glycation of ACE2 receptor and an *in silico* evaluation led to the demonstration that the number of interactions is decreased in comparison to the unmodified model, possibly shifting the virus attack through different, multiple alternative entry routes. Given the evidenced features of this variant, we aimed to investigate with a computational approach the characteristics of Omicron SARS-CoV-2 with respect to its binding to human ACE-2 receptor, in a particular population, namely people affected by diabetes mellitus, at risk for unfavorable outcomes of the COVID-19. The computational analysis, considering the case in which all the lysine residues in the system are subjected to non-enzymatic glycation, confirmed that lysine glycation causes a general loss of interactivity between wild-type (WT)-Spike-RBD and ACE2. In the Omicron variant, Lys417 mutates into an asparagine, preventing the possible non-enzymatic glycation of this residue. Therefore, if non-enzymatic glycation seemed to cause a shift in the way in which the virus enters the cell from the ACE2-mediated mechanism to other pathways, in the case of the Omicron variant the ACE2-mediated approach of the virus seems to remain an important event to take into account. Indeed, interaction profile analysis, together with molecular mechanics–generalized Born surface area (MM-GBSA) calculations, suggests that the Omicron-Spike-RBD maintains a higher affinity for ACE2 subsequently to non-enzymatic glycation with respect to WT-Spike-RBD. The finding of the present computational study may suggest a different clinical relevance of the Omicron variant for the diabetes mellitus field, also in the possible direction of a lower severity of the disease.

## Introduction

**Figure 1:**
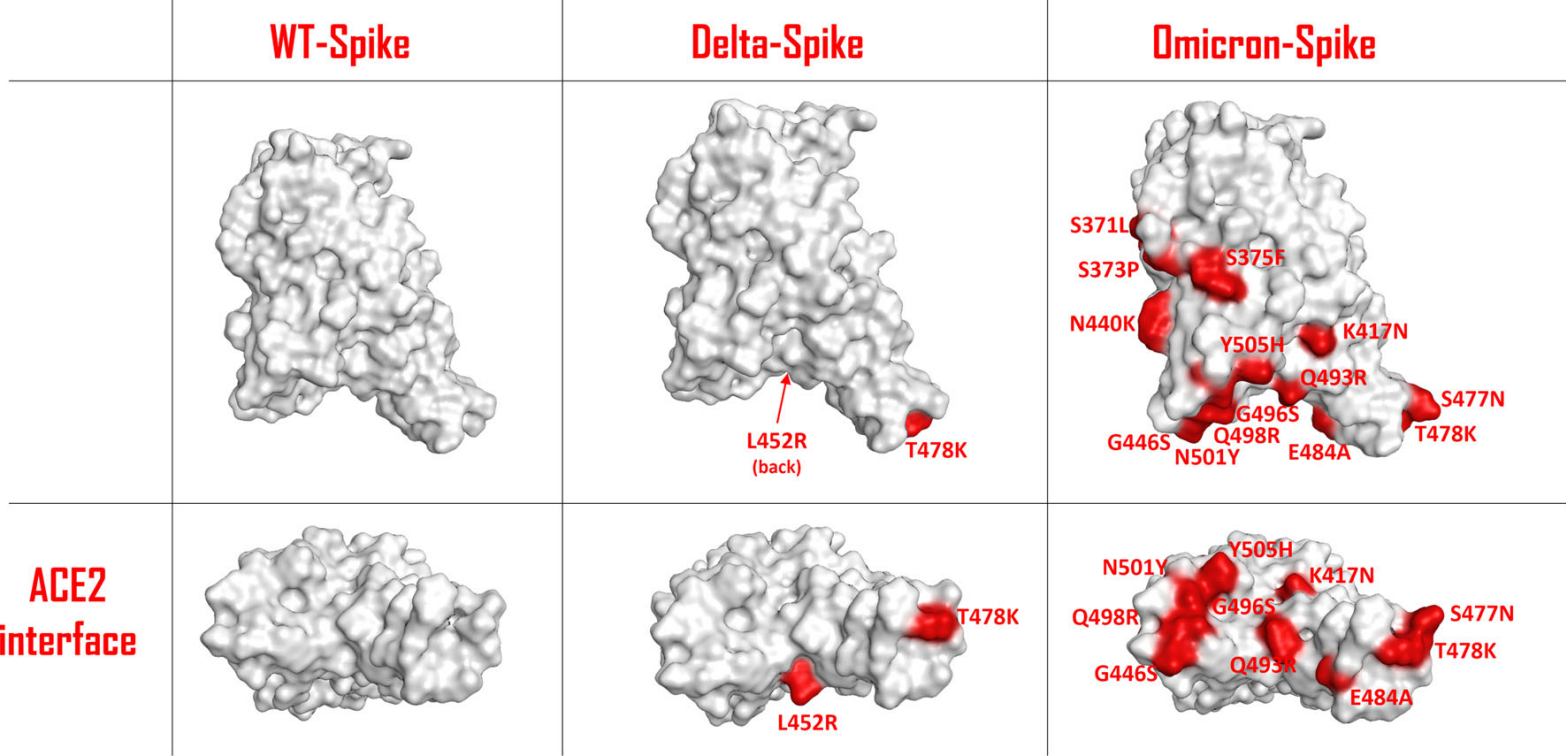
Schematic representation of the SARS-COV-2 Spike protein receptor-binding domain (RBD) surface for the wild type (WT), the Delta variant and the Omicron variant. The residues involved in mutations are colored in red and are also labelled. The top panels represent a lateral view of the Spike-RBDs considered, while the bottom panels highlight the protein surface facing the ACE2 interaction interface for each situation examined.

The unprecedented occurrence of a set of several genetic mutations in the Omicron variant of the SARS-CoV-2 virus has drawn particular attention within scientists and media. Omicron-Spike mutant B.1.1.529 carries more than 30-point mutations in its structure, of which 15 are localized in the receptor-binding domain (RBD) (1). Significant changes have occurred, also with respect to the Delta-Spike variant (**Figure 1**). Moreover, 11 of these mutations are located at the contact surface with ACE2, allowing us to hypothesize that Omicron-Spike could have a relevant change in interactivity with ACE2. Some of these mutations, such as Q498R and N501Y, have already proven to lead to an increased affinity with ACE2 in respect to WT-Spike (2).

The Technical Advisory Group on SARS-CoV-2 Virus Evolution (TAG-VE) has advised WHO that the Omicron variant should be designated as a variant of concern (VOC), due to the epidemiological parameters initially reported in South Africa, and now spreading around the world (3). This is the fifth VOC to be reported since the beginning of the pandemic (4); following the experience with the previously reported variants associated with new worsening of the pandemic, a great concern has arisen whether a relevant change in transmissibility and binding affinity is to be expected with the new Omicron variant. Moreover, most recent data suggest that mutations occurring at RBD influence negatively the activities of neutralizing antibodies induced by vaccines, or administered as monoclonal antibody therapy (5). However, preliminary observations from South Africa suggest that the SARS-CoV-2 Omicron variant is linked to a reduced risk of severe disease when compared to the Delta variant (6, 7).

Since the first times of the pandemic, increased morbidity has been evidenced in people affected by diabetes mellitus (8–11), and the reason for this phenomenon is at present still debated. In a previous report, we hypothesized that the worse outcome of the COVID-19 disease in diabetes mellitus condition could be related to the non-enzymatic glycation of ACE2 receptor, due to hyperglycemic environment, triggering a higher interaction with virus Spike protein (12). However, a specific *in silico* evaluation of the interaction between ACE2 receptor and SARS-CoV-2 Spike protein under different conditions of non-enzymatic glycation has led to the demonstration that the number of interactions is decreased in comparison to the unmodified model (13), possibly shifting the virus attack through different, multiple alternative entry routes. In particular, the interaction of SARS-CoV-2 with human cells has been suggested also be mediated by Transmembrane Protease Serine-2 (TMPRSS2) activity (14, 15), as well as by other receptor pathways, such as Neuropilin-1 (NRP1) (14, 16), dipeptidyl peptidase 4 (DPP4) also known as cluster of differentiation 26 (CD26) (14, 17), the transmembrane glycoprotein CD147 (basigin 2) (14), and glucose-regulated protein 78 (GRP78) (18). However, the role of these alternative routes of virus entry in diabetes is still a matter of debate and investigation (13).

In view of the evidenced features of this variant, we aimed to investigate with a computational approach the characteristics of Omicron SARS-CoV-2 with respect to its binding to human ACE-2 receptor, in a particular population, namely people affected by diabetes mellitus, at risk for unfavorable outcomes of the COVID-19. The *in silico* analysis, after the recognition of the main interactions occurring between virus and ACE2 receptor, was directed to the specific evaluation of the impact to the affinity of the system, induced by a hyperglycemic environment, conditioning non-enzymatic glycation at lysine residues of both Omicron-Spike-RBD and ACE2 receptor.

## Materials and Methods

The computational analysis presented in this study was conducted starting from two different experimental structures, which were both downloaded from the Protein Data Bank (PDB) (19). The first of these systems represents the wild-type (WT) form of SARS-CoV-2 Spike RBD complexed with ACE2 receptor (PDB code: 6M0J; method: X-ray diffraction; resolution: 2.45 Å) (20), while the second involves the SARS-CoV-2 Spike-RDB Omicron variant forming a complex with ACE2 (PDB code: 7T9L; method: cryo-electron microscopy (Cryo-EM); resolution: 2.66 Å) (21). The proteins were prepared for molecular modeling with the “Structure Preparation” tool implemented in the Molecular Operating Environment (MOE) suite (22). The missing hydrogen atoms were added exploiting the MOE “Protonate 3D” tool, which assigns to each residue the most probable protonation state at the chosen pH, which in our case was set at the value of 7.4. These hydrogen atoms were then minimized using the AMBER10:EHT (23) force field implemented in MOE. To create the glycated forms of both WT-Spike-RBD/ACE2 and Omicron-Spike-RBD/ACE2 systems, the lysine residues of the prepared complexes 6M0J and 7T9L were manually changed. This operation involved the addition of a cyclic amino sugar moiety, which results from the Maillard reaction between the lysine amino acid and D-glucose, followed by the Amadori rearrangement (24) (as depicted in **Supplementary Figure 1**). This step allowed us to obtain the four systems of interest for our study, considering various conditions of non-enzymatic glycation (here labeled as “glyco”). These complexes are WT-Spike-RBD with ACE2, WT-Spike-RBD-glyco with ACE2-glyco, Omicron-Spike-RBD with ACE2, and Omicron-Spike-RBD-glyco with ACE2-glyco. Once the different systems were obtained, the glycated lysine residues were energetically minimized under the AMBER10:EHT (23) force field implemented in MOE. This passage was essential in order to allow the newly introduced sugar groups to be properly orientated in the environment.

After these preliminary steps, the interactions between Spike-RBD and ACE2 were analyzed both visually and with the “GetContacts” tool (25). This last program can extrapolate and classify all the interactions between the biological entities of a system. In our case, the specific contacts between Spike-RBD and ACE2 were evaluated for all the cases.

**Figure 2:**
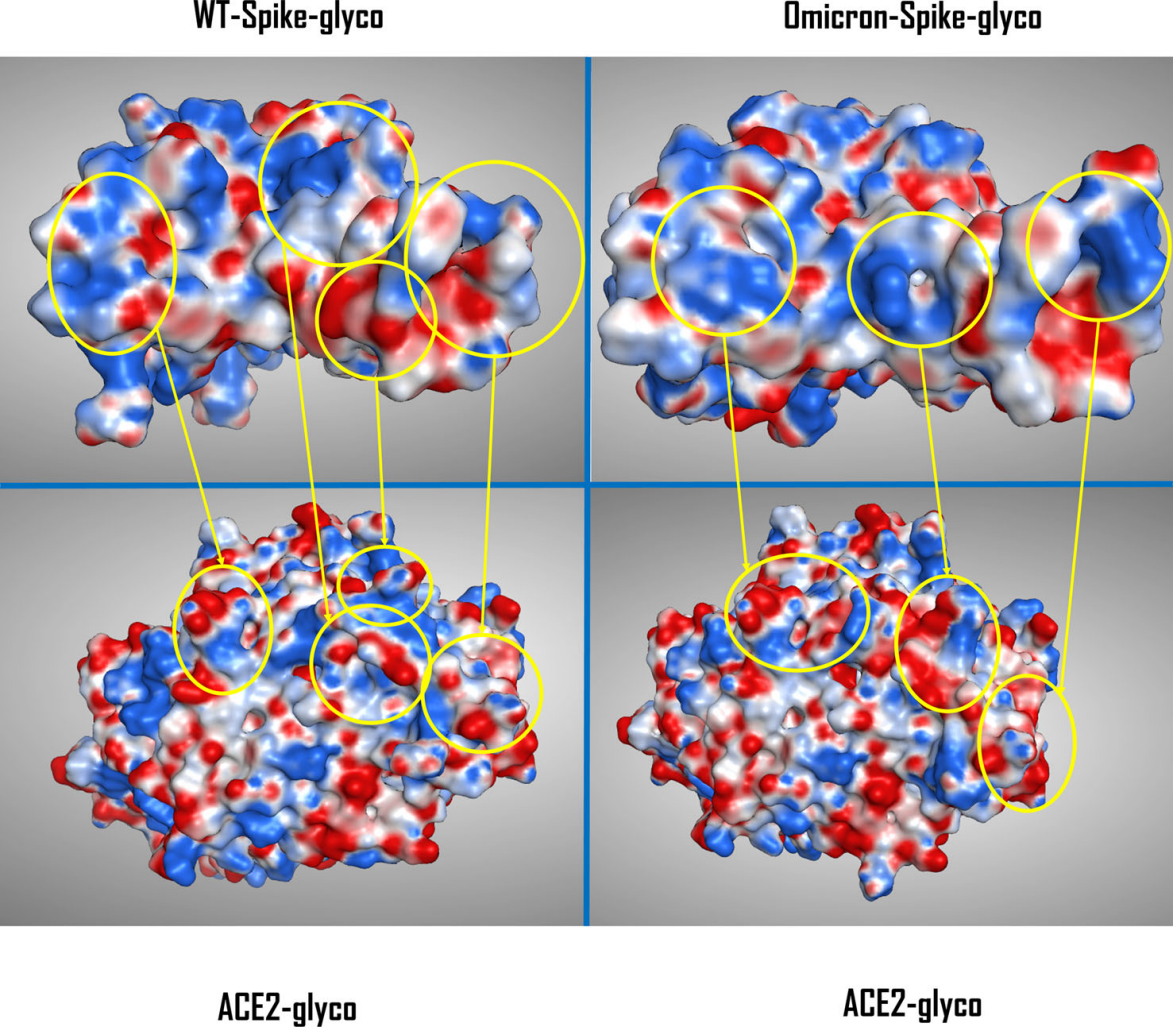
Comparison between the electrostatic contact surface between the WT-Spike-RBD-glyco/ACE2-glyco system (top and bottom left) and the Omicron-Spike-RBD-glyco/ACE2-glyco complex (top and bottom right). The areas of highest variation in electrostatic distribution between glycated WT and Omicron Spike-RBDs are circled in yellow and are then connected with the corresponding ACE2-glyco interacting surface portion. The Spike-RBD/ACE2 backbone conformation for the WT variant comes from the crystal with PDB code 6M0J, while for the Omicron variant the data come from the Cryo-EM structure with PDB code 7T9L. WT, wild type; RBD, receptor-binding domain; PDB, Protein Data Bank; Cryo-EM, cryo-electron microscopy.

To give a better view of the macroscopic changes brought by the mutation and the glycation of the systems, the electrostatic surface of both ACE2 and Spike-RBD were also calculated for all the situations considered (as depicted in **Figure 2**).

To further inspect the effect of glycation on the interaction between Spike-RBD and ACE2 receptor for both WT and Omicron variants, molecular mechanics–generalized Born surface area (MM-GBSA) calculations were executed in the complexes using the Schrödinger Prime application (26). This method consists of the estimation of the binding free energy between two entities in an environment, exploiting an implicit solvation model. In our case, the Surface Generalized Born Model and Variable Dielectric (VSGB) solvation model (27) was used, and the force field in which the complexes were evaluated was the OPLS4 force field (28) implemented in Prime.

## Results

A visual representation of the complexes created considering various conditions of non-enzymatic glycation on the SARS-CoV-2 Spike RBD/ACE2 receptor system is reported in **Supplementary Figures 2**–**5**. A brief report of the number of the interactions (expressed in terms of the number of pairs of residues that are in contact between the two proteins) in each system is reported in **Table 1**.

**Table 1:** Number of interactions (expressed in terms of the number of residues pairs that are in contact between the two proteins) extrapolated from the four systems considered in the study presented (WT-Spike-RBD on ACE2, WT-Spike-RBD-glyco on ACE2-glyco, Omicron-Spike-RBD on ACE2, and Omicron-Spike-RBD-glyco on ACE2-glyco).

Interaction type	WT-Spike-RBD on ACE2	WT-Spike-RBD-glyco on ACE2-glyco	Omicron-Spike-RBD on ACE2	Omicron-Spike-RBD-glyco on ACE2-glyco
**Salt bridge interactions**	1	0	2	1
**Hydrogen bonds**	9	10	11	10
**van der Waals interactions**	30	21	32	37

WT, wild type; RBD, receptor-binding domain.

The“GetContacts” tool was exploited in order to calculate all the contacts between Spike and ACE2 in each of the scenarios considered.

The interaction pattern occurring in the native WT-Spike-RBD/ACE2 system (**Supplementary Figure 2**) is characterized by polar bonds (comprising both salt bridges and hydrogen bonds) as well as non-polar van der Waals interactions. The WT-Spike-RBD-glycated/ACE2-glycated system (**Supplementary Figure 3**), mimicking the maximum level of glycation attainable in a hyperglycemic condition in the case of native SARS-CoV-2, is characterized by a reduction in the number of interactions, mainly of a non-polar entity.

The effect of glycation on the new Omicron SARS-CoV-2 variant (compare **Supplementary Figure 4** and **Supplementary Figure 5**), produces a diminished number of bonds as well, occurring in virus/receptor interaction. As depicted from the results obtained, while for the WT-Spike-RBD/ACE2 situation the reduction in the number of non-polar interactions due to glycation is not linked to an overall change in the number of polar contacts, for the Omicron variant, the small decrease in the number of hydrogen bonds subsequent to the non-enzymatic glycation of lysine amino acids seems to be compensated by an increase in non-polar interactions.

Consequently, we can hypothesize that, if the decrement in non-polar contacts between WT-Spike-RBD/ACE2 and WT-Spike-RBD-glyco/ACE2-glyco systems is not balanced by a significant strengthening of the polar component of the interaction, for the Omicron variant, where the number of polar bonds is higher with respect to the WT in both native and glycated forms, the interaction between the two glycated proteins could be more efficiently preserved. This is also supported by the MM-GBSA calculation results, reported in **Table 2**, which highlight the loss in interaction energy that both the WT-Spike-RBD/ACE2 and the Omicron-Spike-RBD/ACE2 complexes experienced after glycation. The data coming from this analysis confirm that Omicron-Spike-RDB is able to form with ACE2 a more stable complex in respect to the WT-Spike-RBD (with a difference in the binding free energy of about 11 kcal/mol). Moreover, the binding free energies obtained demonstrate that glycation causes a general loss in interactivity towards ACE2 for both for WT-Spike-RBD and Omicron-Spike-RBD, and that this drop is much more significant for the WT variant.

**Table 2:** Free energy of binding (expressed in kcal/mol) obtained with the MM-GBSA calculations executed on the different complexes considered in this study (WT-Spike-RBD/ACE2, WT-Spike-RBD-glyco/ACE2-glyco, Omicron-Spike-RBD/ACE2, and Omicron-Spike-RBD-glyco/ACE2-glyco).

Interaction type	WT-Spike-RBD on ACE2	WT-Spike-RBD-glyco on ACE2-glyco	Omicron-Spike-RBD on ACE2	Omicron-Spike-RBD-glyco on ACE2-glyco
**MM-GBSA total free energy of binding (kcal/mol)**	-114.37	-89.29	-125.59	-111.83

MM-GBSA, molecular mechanics–generalized Born surface area; WT, wild type; RBD, receptor-binding domain.

**Figure 3:**
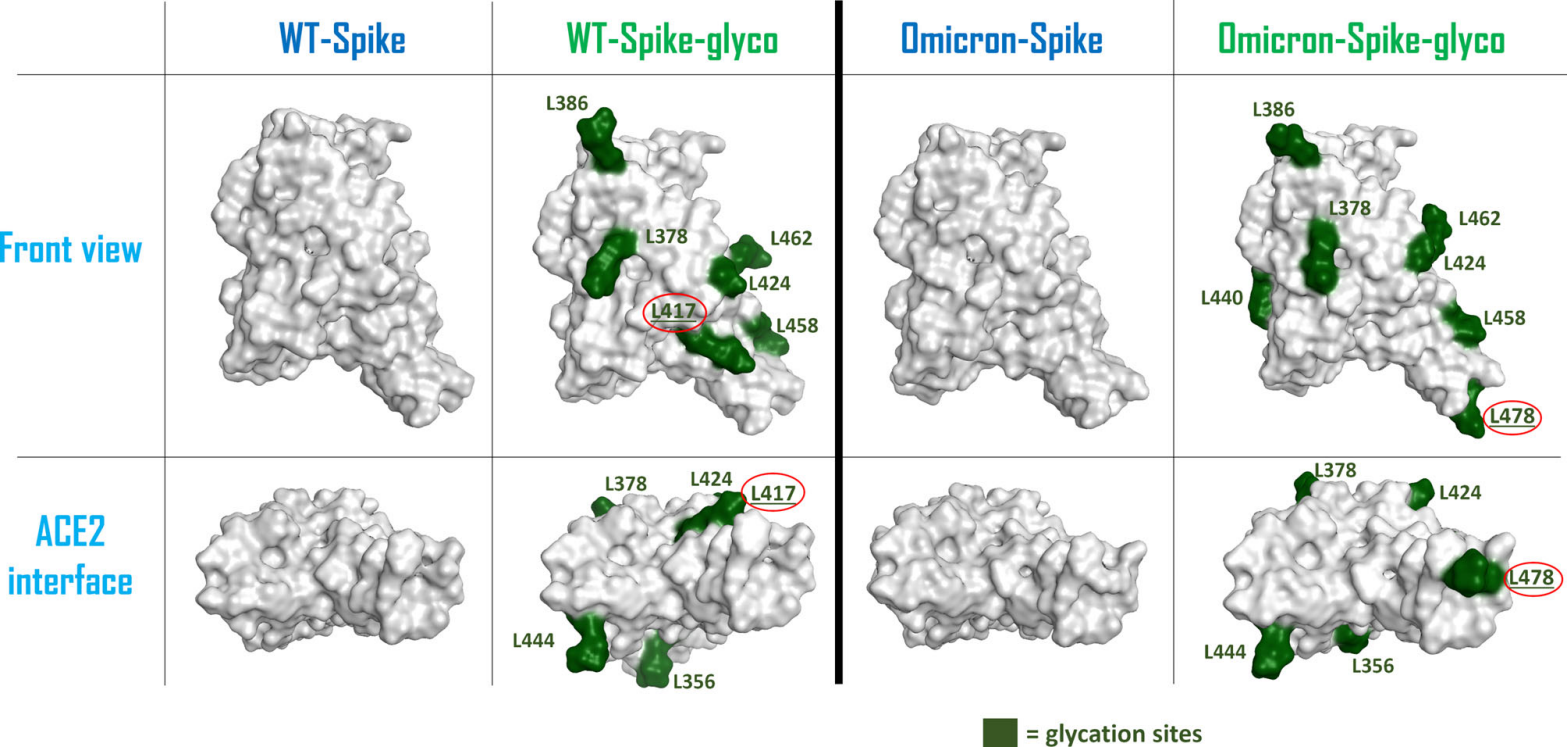
Schematic representation of the surfaces of the possible forms of Spike-RBD considered in this computational study. From the left, the systems considered are: wild-type (WT) Spike-RBD, the lysine-glycated form of wild-type (WT) Spike-RBD, the Omicron variant Spike-RBD and the glycated form of Omicron variant Spike-RBD. For the glycated systems, the surface of lysine amino acids has been colored in dark green and has also been labeled. The lysine which are located on the contact interface with ACE2 (Lys417 for the WT-Spike-RBD-glyco and Lys478 for the Omicron-Spike-RBD-glyco) have their label underlined and circled in red. The top figures represent a lateral view of the Spike-RBDs considered, while the bottom figures highlight the protein surface facing the ACE2 interaction interface for each situation examined. RBD, receptor-binding domain.

To allow a more comprehensive view of the overall changes caused by the Omicron mutation and the glycation of the systems, the electrostatic surface of both ACE2 and Spike-RBD were calculated for all the situations considered, and the differences between the electrostatic distribution were highlighted (**Supplementary Figure 6** and **Figure 2**). As illustrated by these pictures, it is interesting to notice the linkage between the change in the surface features of the proteins and the locations in which glycation takes place. To represent this parallelism, the glycation sites were highlighted on the Spike-RBD surface, and the results are reported in **Figure 3**. As depicted, the only Spike-RBD lysine residue directly in contact with ACE2 is Lys417 in the native form. Although in the Omicron variant this lysine mutates into an arginine, Thr478 mutates into lysine (T478K), forming another glycation site on the ACE2 interface.

## Discussion

The computational analysis allowed us to evaluate the changes in the interaction pattern between Spike-RBD and ACE2 receptor considering both mutation (focusing on the Omicron variant) and the presence of a hyperglycemic environment. The modified affinity between these two biological entities, which seems higher for Omicron-Spike-RBD to WT-Spike-RBD, has been reported (21, 29, 30). In a hyperglycemic environment, typical of diabetes, we considered the case in which all the lysine residues in the system are subjected to non-enzymatic glycation. The behavior of the proteins will so change with respect to the position of the lysine amino acids. A recent work of our group highlighted the possibility that diabetic patients in which this glycation process takes place are less prone to be infected by SARS-CoV-2 in an ACE2-dependent way (13). Indeed, our analysis here confirms the result and suggests that lysine glycation causes a general loss of interactivity between WT-Spike-RBD and ACE2.

One of the main WT-Spike-RBD residues involved in the interaction with ACE2 is Lys417, which is the lysine amino acid that is most exposed to the ACE2 interface when Spike-RBD approaches the host cells. Indeed, glycation at this residue could lead to a weakening of the contacts between the two entities, mainly due to the loss of the strong salt bridge between the positive-charged nitrogen of Spike-RBD Lys417 and the negative-charged oxygen of ACE2 Asp30. Moreover, the increased hindrance of the glycated moiety at the contact interface can contribute to a general decrease of the interaction strength between Spike-RBD and ACE2 residues at the interface.

In the Omicron variant, Lys417 mutates into an asparagine, preventing the possible non-enzymatic glycation of this residue. This variation, together with several mutations on Spike-RBD that were linked to an increased affinity for ACE2 (such as S477N, Q498R, and N501Y) (31), is predicted to contribute to an increase in the interactivity between Omicron-Spike-RBD and ACE2 in a hyperglycemic environment. This behavior is shown by **Supplementary Figures 2**–**5**, in which the number of contacts between Spike-RBD and ACE2 (derived from the calculation executed with the “GetContacts” tool) is reported for the different systems considered. **Table 1** summarizes the overall number of residues pairs in contact between each kind of Spike-RBD/ACE2 molecular complex. First, as mentioned, both interaction analysis and MM-GBSA calculations highlight the higher affinity that Omicron exerts for ACE2 with respect to the WT variant. The data coming from the MM-GBSA calculation show that the reduction in the number of contacts between WT-Spike-RBD and ACE2 subsequent to glycation causes an increase in the binding free energy which is more significant than the one experienced by Omicron-Spike-RBD. The glycation, as expected, causes in both WT and Omicron variants a reduction in interaction strength with ACE2 receptor, and this can be attributed to different molecular reasons. For WT-Spike-RBD, the glycation causes mainly a loss in non-polar interaction, which could be also associated with a loss in the efficiency of hydrogen-bonding, leading to an overall noticeable increase in binding free energy. In the case of Omicron-Spike-RBD, where the loss in the number of polar contacts is compensated by an increase in non-polar interaction, and the number of non-polar contacts not only does not decrease, but also increases, the loss in binding free energy seems to be fully attributable to a drop in the efficiency of hydrogen bonding. Indeed, the increased hindrance created by the glycated residues present on the Omicron-Spike-RBD-glyco/ACE2-glyco interface (e.g., ACE2 Lys353) could destabilize the web of polar contacts that, even if the number is retained, decrease in strength. In any case, the data coming both from the analysis of the number of interactions and from MM-GBSA calculations highlight higher stability of the Omicron-Spike-RBD-glyco/ACE2-glyco complex over the WT-Spike-RBD-glyco/ACE2-glyco system.

The results of our analysis allow us to hypothesize that the affinity between the viral protein Spike and the human receptor ACE2 is higher for the Omicron variant in respect to the WT both in native conditions and also in the case of non-enzymatic glycation, typical of the hyperglycemic environment.

The results of the computational analysis here conducted allow us to hypothesize that, if non-enzymatic glycation seemed to cause a shift in the way the virus enters the cell from the ACE2-mediated mechanism to other pathways, in the case of the Omicron variant the ACE2-mediated approach of the virus seems to remain an important event to take into account. Indeed, both our number and our MM-GBSA analysis suggest that, even if a loss in interactivity is noticeable between the Omicron-Spike-RBD/ACE2 and the Omicron-Spike-RBD-glyco/ACE2-glyco, these systems are able to maintain a higher affinity with each other in respect to, respectively, the WT-Spike-RBD/ACE2 and WT-Spike-RBD-glyco/ACE2-glyco complexes.

The finding of the present computational study may suggest several consequences of potential clinical relevance for the diabetes mellitus field.

Non-enzymatic glycation is a well-documented phenomenon occurring in diabetes, and the fast kinetics of the event has been proven. Atanasova et al. (32) demonstrated that non-enzymatic glycation of protein amino groups (Maillard reaction) can occur at high glucose concentrations very quickly, already after few minutes. Therefore, a non-enzymatic glycation of ACE2 receptor in target tissues exposed to virus entry is very likely a fast spreading-out process, and the virus Spike protein can be glycated as well very quickly when high glucose concentrations occur.

If on one side the glycation might prevent the ACE2-Spike interaction in diabetes mellitus, as previously suggested (13), on the other side the mutations of the Omicron-variant Spike protein, leading to the loss of a lysine glycation site, may be responsible for a relatively augmented binding affinity to ACE2 (compare with **Figure 3**). Therefore, the risk of a more pronounced virus binding to ACE-2 receptor may occur in patients with diabetes, possibly conditioning a higher susceptibility to SARS-CoV-2 infection, as indicated in our first hypothesis (12). Moreover, in patients with decompensated diabetes, the greater affinity of Omicron Spike for ACE2 could on the one hand enhance the contact with the virus and therefore infection rate, but on the other hand, may reduce the possibility of entry through alternative routes and thus modify the overall course and severity of the disease. The new suggested roles of a novel furin cleavage site, exploited by SARS-CoV-2 to become fully active (33), could represent a possible escape mechanism used by the virus to produce an infection that, in the case of diabetes mellitus, may assume a pernicious evolution. The possible occurrence of alternative ways of virus entry in people affected by diabetes, as recently suggested (13), makes the new Omicron virus mutation of peculiar interest, not only in the general population, but also particularly in diabetes and pre-diabetes areas. However, the present results seem to point towards a lower severity of the disease with the Omicron variant also for patients with diabetes mellitus, supporting very recently published data (7), which show that diabetes did not appear to be a co-morbidity factor influencing disease severity, but only age. The severity of COVID-19 in diabetes remains a clinical query, also because the overall course of the infectious disease is influenced by a pre-existing reactivity, characterized by an increased pro-inflammatory profile (34), also linked to an excess of adipose tissue, which is associated with augmented lymphocyte activation and cytokine production (35). Whether this new variant may influence the real-world clinical evolution of COVID-19 in people affected by diabetes mellitus is still a matter of speculation. As suggested by the comment of Karim and Karin (4), we await knowledge of how this new variant will develop.

## Data Availability Statement

The original contributions presented in the study are included in the article/**Supplementary Material**. Further inquiries can be directed to the corresponding author.

## Author Contributions

DB, ER, GS, and SM contributed to the conception and design of the research. DB and SM performed the molecular modeling experiments. DB, ER, SM, and GS drafted the paper. AL and SM supervised and edited the manuscript. All authors contributed to the article and approved the submitted version.

## Funding

This research work was funded by the University of Padua (Italy).

## Conflict of Interest

The authors declare that the research was conducted in the absence of any commercial or financial relationships that could be construed as a potential conflict of interest.

## Publisher’s Note

All claims expressed in this article are solely those of the authors and do not necessarily represent those of their affiliated organizations, or those of the publisher, the editors and the reviewers. Any product that may be evaluated in this article, or claim that may be made by its manufacturer, is not guaranteed or endorsed by the publisher.
