# Supplementary material for: Omicron Variant of SARS-CoV-2 Virus: In Silico Evaluation of the Possible Impact on People Affected by Diabetes Mellitus
Source: Front Endocrinol (Lausanne). 2022 Mar 7;13:847993. doi: 10.3389/fendo.2022.847993 (PMC8935058; doi:10.3389/fendo.2022.847993)
Supplement: Supplementary file 1 [file DataSheet_1.pdf]

Supplementary Material

Omicron variant of SARS-CoV-2 virus: *in silico* evaluation of the possible impact on people affected by diabetes mellitus

Davide Bassani[1], Eugenio Ragazzi[*2], Annunziata Lapolla[3], Giovanni Sartore[3], Stefano Moro[1]

[1]Department of Pharmaceutical and Pharmacological Sciences (DSF), Molecular Modeling Section (MMS), University of Padova School of Medicine and Surgery, Via Marzolo, 5, 35131 Padua, Italy.
[2]Department of Pharmaceutical and Pharmacological Sciences (DSF), University of Padova School of Medicine and Surgery, Largo Meneghetti 2, 35131 Padua, Italy.
[3]Department of Medicine (DIMED), University of Padova School of Medicine and Surgery, Via Giustiniani 2, 35128 Padua, Italy.

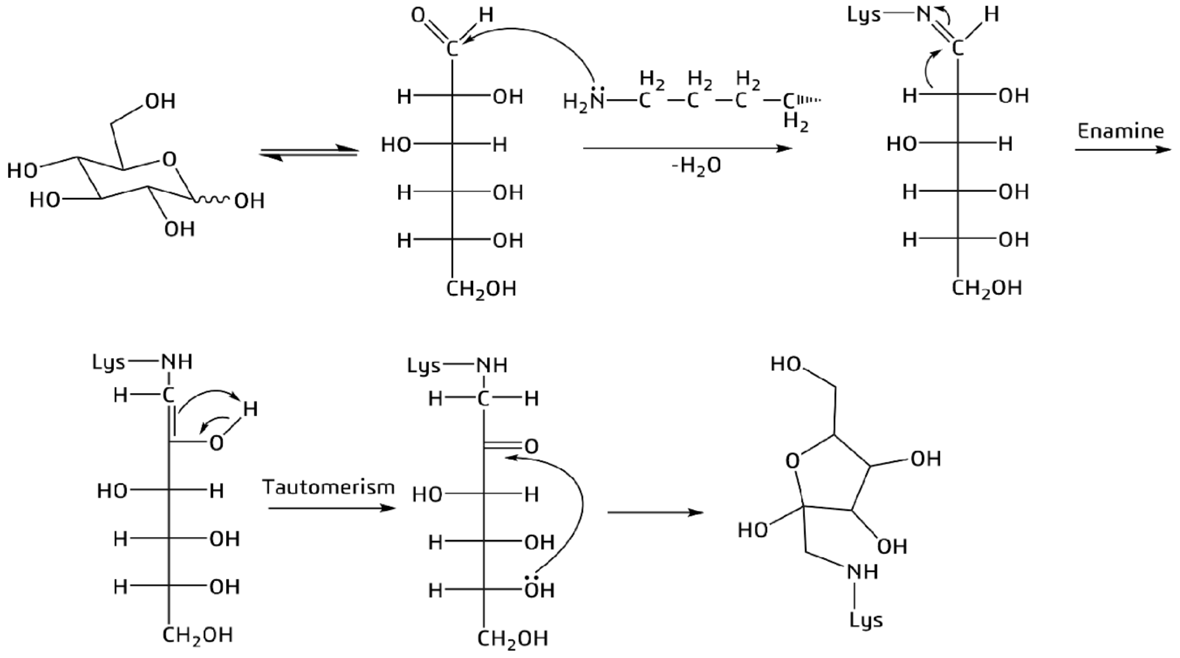

Supplementary Figure 1. Schematic representation of the mechanism of non-enzymatic glycation of a lysine amino acid residue by D-glucose. As shown, the final product is the cyclic amino sugar that we considered for the study.

WT-Spike-native/ACE2-native

Type of interaction	Number of contacts
salt bridge	1
hydrogen bond	9
van der Waals interaction	30

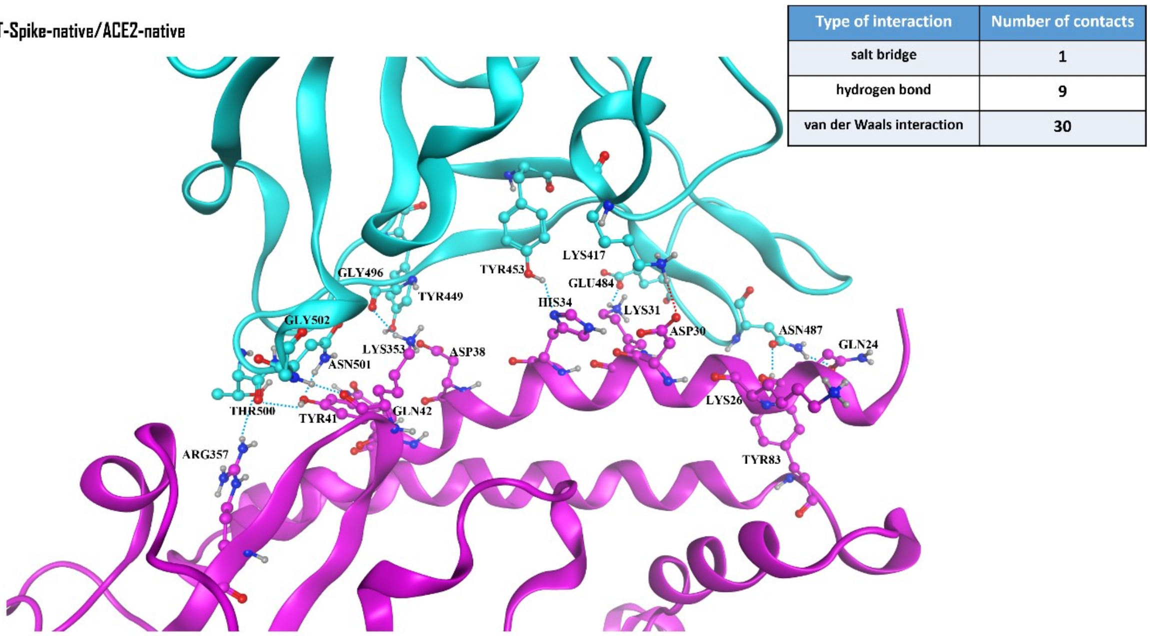

Supplementary Figure 2. Representation of the WT-Spike-RBD/ACE2 complex coming from the crystal structure 6M0J. The table on the upper right lists the number of the interactions between the two proteins (expressed as the number of couples of residues interacting with each other). The hydrogen bonds are highlighted with dashed lines colored in cyan, while the salt-bridge interaction is shown with the red dashed line.

WT-Spike-glyco/ACE2-glyco

Type of interaction	Number of contacts
salt bridge	0
hydrogen bond	10
van der Waals interaction	21

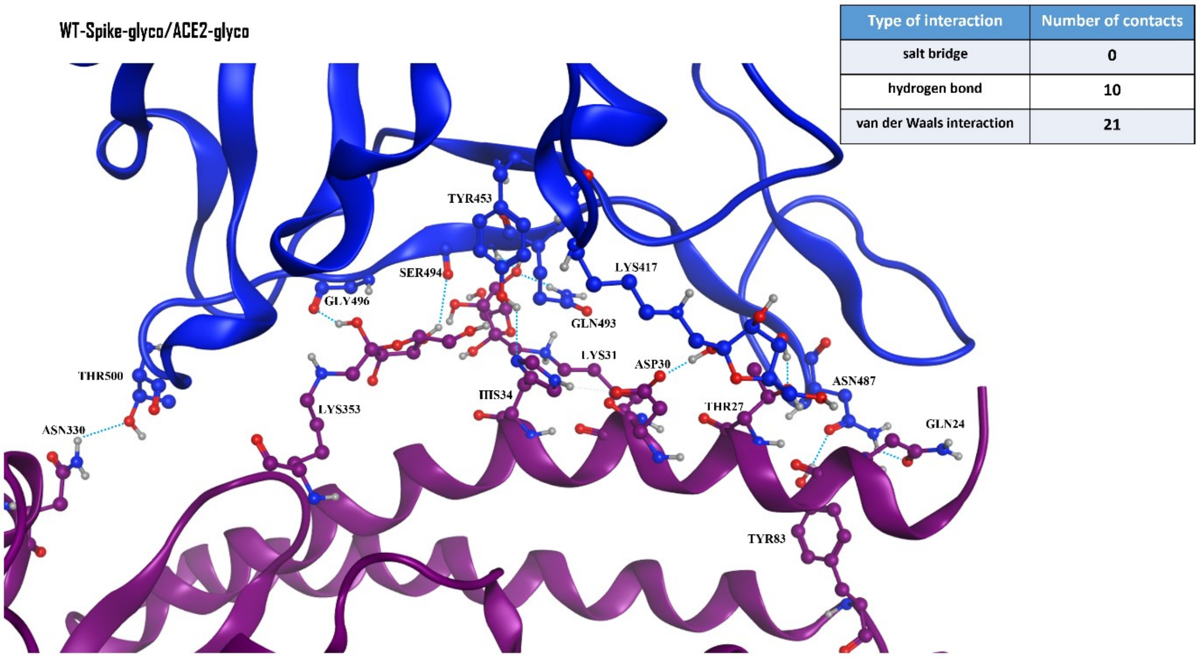

Supplementary Figure 3. Representation of the WT-Spike-RBD-glyco/ACE2-glyco complex. The structure is derived from the manual addition of the sugar moieties on each lysine amino acid of the crystal structure 6M0J, followed by their energetic minimization. The table on the upper right lists the number of the interactions between the two proteins (expressed as the number of couples of residues interacting with each other). The hydrogen bonds are highlighted with dashed lines colored in cyan.

Omicron-Spike-native/ACE2-native

Type of interaction	Number of contacts
salt bridge	2
hydrogen bond	11
van der Waals interaction	32

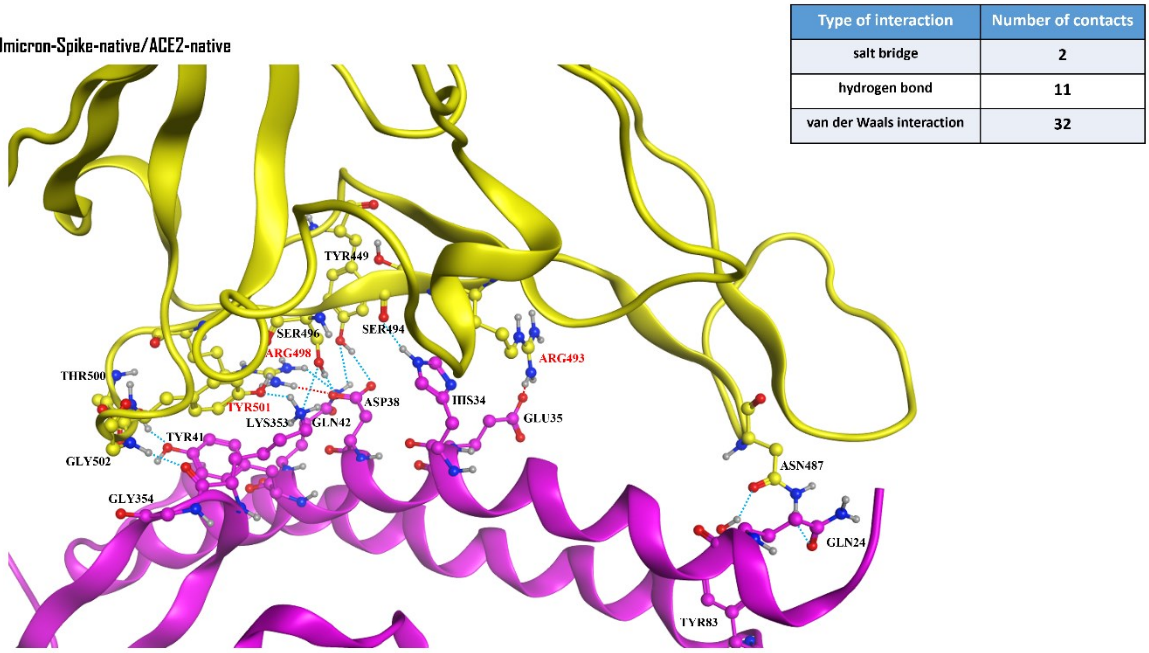

Supplementary Figure 4. Representation of the Omicron-Spike-RBD/ACE2 complex coming from the Cryo-EM structure 7T9L. The table on the upper right lists the number of the interactions between the two proteins (expressed as the number of couples of residues interacting with each other). The hydrogen bonds are highlighted with dashed lines colored in cyan, while the salt-bridge interactions are shown with the red dashed lines.

Omicron-Spike-glyco/ACE2-glyco

Type of interaction	Number of contacts
salt bridge	1
hydrogen bond	10
van der Waals interaction	37

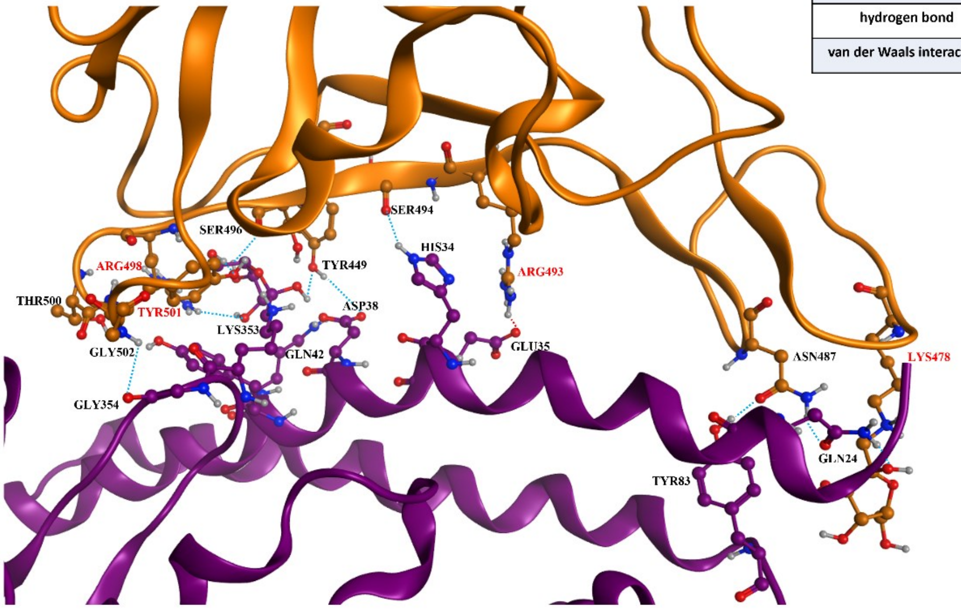

Supplementary Figure 5. Representation of the Omicron-Spike-RBD-glyco/ACE2-glyco complex. The structure is derived from the manual addition of the sugar moieties on each lysine amino acid of the Cryo-EM structure 7T9L, followed by their energetic minimization. The table on the upper right lists the number of the interactions between the two proteins (expressed as the number of couples of residues interacting with each other). The hydrogen bonds are highlighted with dashed lines colored in cyan, while the salt-bridge interaction is shown with the red dashed line.

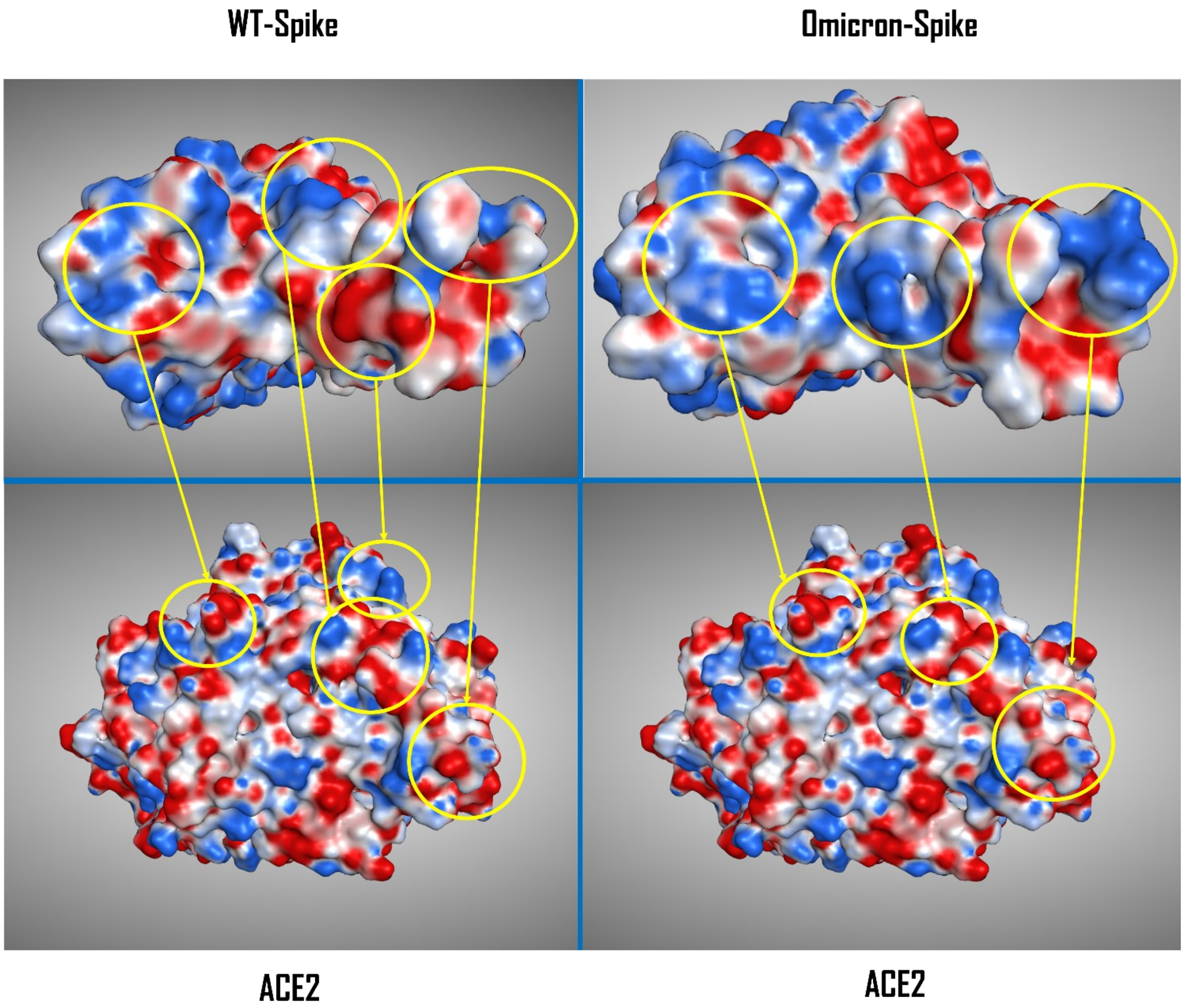

Supplementary Figure 6. Comparison between the electrostatic contact surface between the WT-Spike-RBD/ACE2 system (upper and lower left) and the Omicron-Spike-RBD-glyco/ACE2-glyco complex (upper and lower right). The areas of highest variation in electrostatic distribution between WT and Omicron Spike-RBDs are circled in yellow and are then connected with the corresponding ACE2-interacting surface portion. The Spike-RBD/ACE2 conformation for the WT variant comes from the crystal with PDB code 6M0J, while for the Omicron variant the data come from the Cryo-EM structure with PDB code 7T9L.
